# A rare case of pycnodysostosis during pregnancy

**DOI:** 10.1002/ccr3.6565

**Published:** 2022-11-06

**Authors:** Aisha Alshdefat, Abdulqadir J. Nashwan, Hayat Al Bibi

**Affiliations:** ^1^ Maternal and Child Health Department College of Nursing, Sultan Qaboos University Muscat Oman; ^2^ Nursing Department Hamad Medical Corporation Doha Qatar; ^3^ Al Yarmouk University Irbid Jordan

**Keywords:** cathepsin K, defective gene, dwarfisms, lysosomal storage disease, pycnodysostosis

## Abstract

We describe a case in which a 41‐year‐old pregnant female with pycnodysostosis presented for elective cesarean section at week 37 + 5 of pregnancy. This is the first reported case of pycnodysostosis during pregnancy in Oman, to the author's best knowledge.

## Graphical abstract

The authors urge clinicians to diagnose cases with pycnodysostosis early, especially during pregnancy. The characteristics of pycnodysostosis may make anesthesia management more challenging.

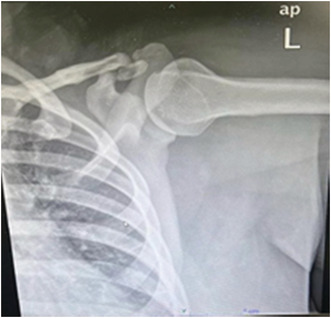

## 1 INTRODUCTION

Pycnodysostosis is an autosomal recessive osteochondrodysplasic disorder that is often identified in childhood. It is categorized as a bone lysosomal storage disorder caused by mutations in the gene encoding the enzyme cathepsin K on chromosome 1, with an estimated frequency of 1–3 per 1,000,000 people.^1^ Historically, pycnodysostosis was discovered by Maroteaux and Lamy in 1962, and the faulty gene was identified in 1996.^2^ The discovery of the faulty gene that causes pycnodysostosis allows for more accurate diagnosis, carrier testing, and knowledge of the illness. Pycnodysostosis is distinguished from other osteochondrodysplasic disorders by low height, skull dysplasia, a flattened mandibular angle, clavicle, and terminal phalange dysplasia, increased bone density, and normal laboratory findings.^3^ These characteristics may complicate anesthetic management. Endotracheal intubation by traditional direct laryngoscopy may be complicated by craniofacial anomalies, which may lead to cervical spine fracture.^4^ Here, we describe a case of a pregnant lady with pycnodysostosis presented for an elective cesarean section.

## 2 CASE PRESENTATION

**FIGURE 1:**
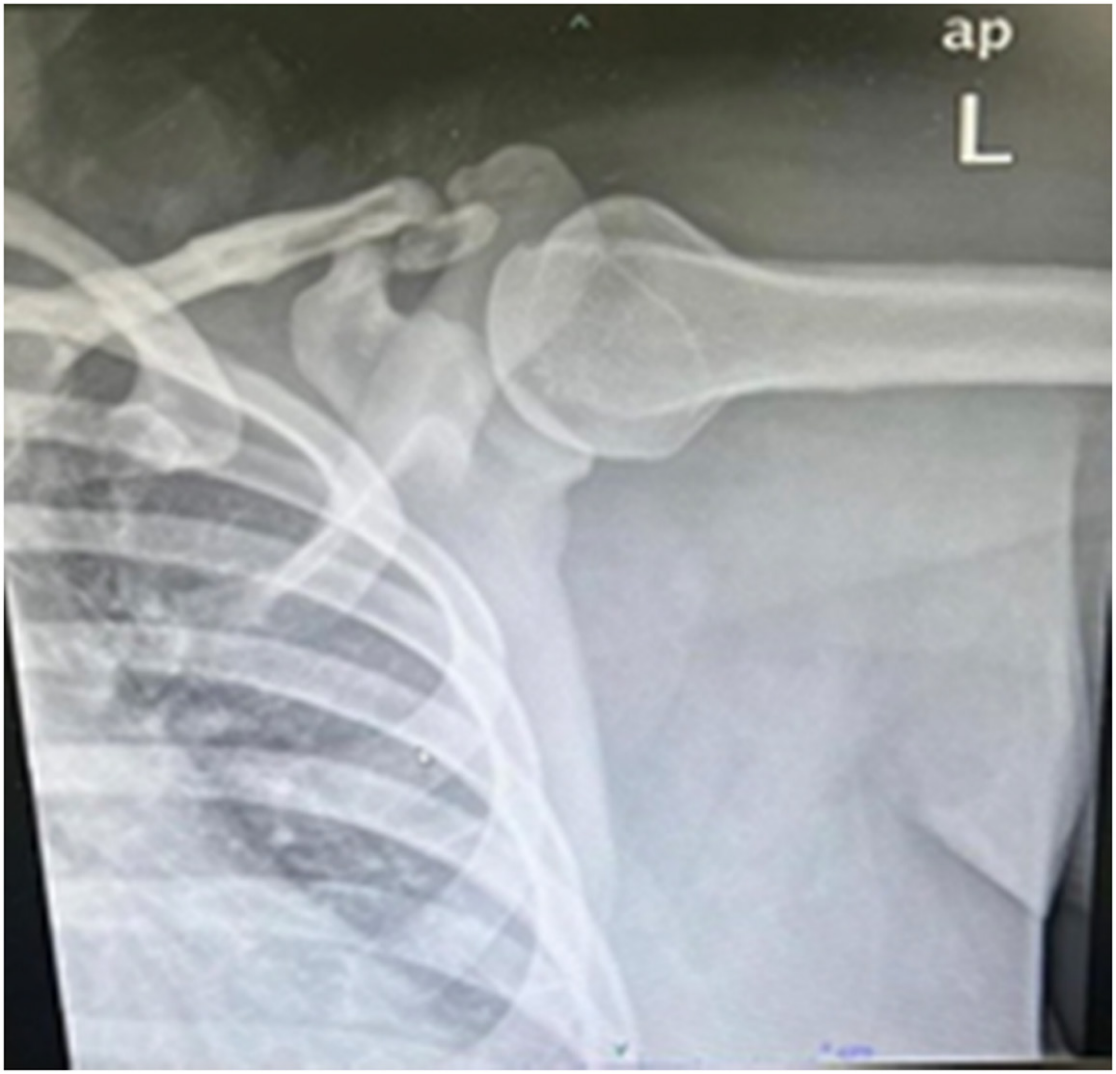
Left Shoulder X‐Ray AP Coracoid process shows fracture.

A 41‐year‐old woman (G3P1A1) diagnosed with pycnodysostosis was scheduled for an elective cesarean delivery at week 37 + 5 of pregnancy. She had a history of previous cesarean sections done before 10 years for breach presentation and non‐reassuring CTG, second pregnancy complete miscarriage at 12 weeks, and current pregnancy, spontaneous conception with gestational diabetes. She was unfit for a spinal anesthesia due to labor pain and the decision was to go for general anesthesia. General anesthesia was challenging during intubation and post‐extubation; the patient developed an aspiration pneumonia (chest x‐ray showed infiltrate to left side) (See Figure 1) and managed with antibiotics without admission to the intensive care unit (ICU).

The patient was 140 cm in height, weighed 60 kg, and had a Body Mass Index (BMI) of 35.9. She has a short neck, small mandible, and mallampati classification (MPC), thyromental distance (TMD). She had known allergies to butamirate® (cough suppressant) and rhinopro® (pseudoephedrine & carbinoxamine) and was not taking any home medications.

Laboratory investigation reveal blood group O‐Rh positive, Hb 11.8 g/dl, platelets 308 platelets per microliter, HIV negative, OGTT 5.4/8.7 mmoL/L, sickling negative, no anomaly scan, polyhydramnios DVP 8.3 c. vital signs were pulse 108/min, Bp 110/58 mmhg, Spo2 98%.

**FIGURE 2:**
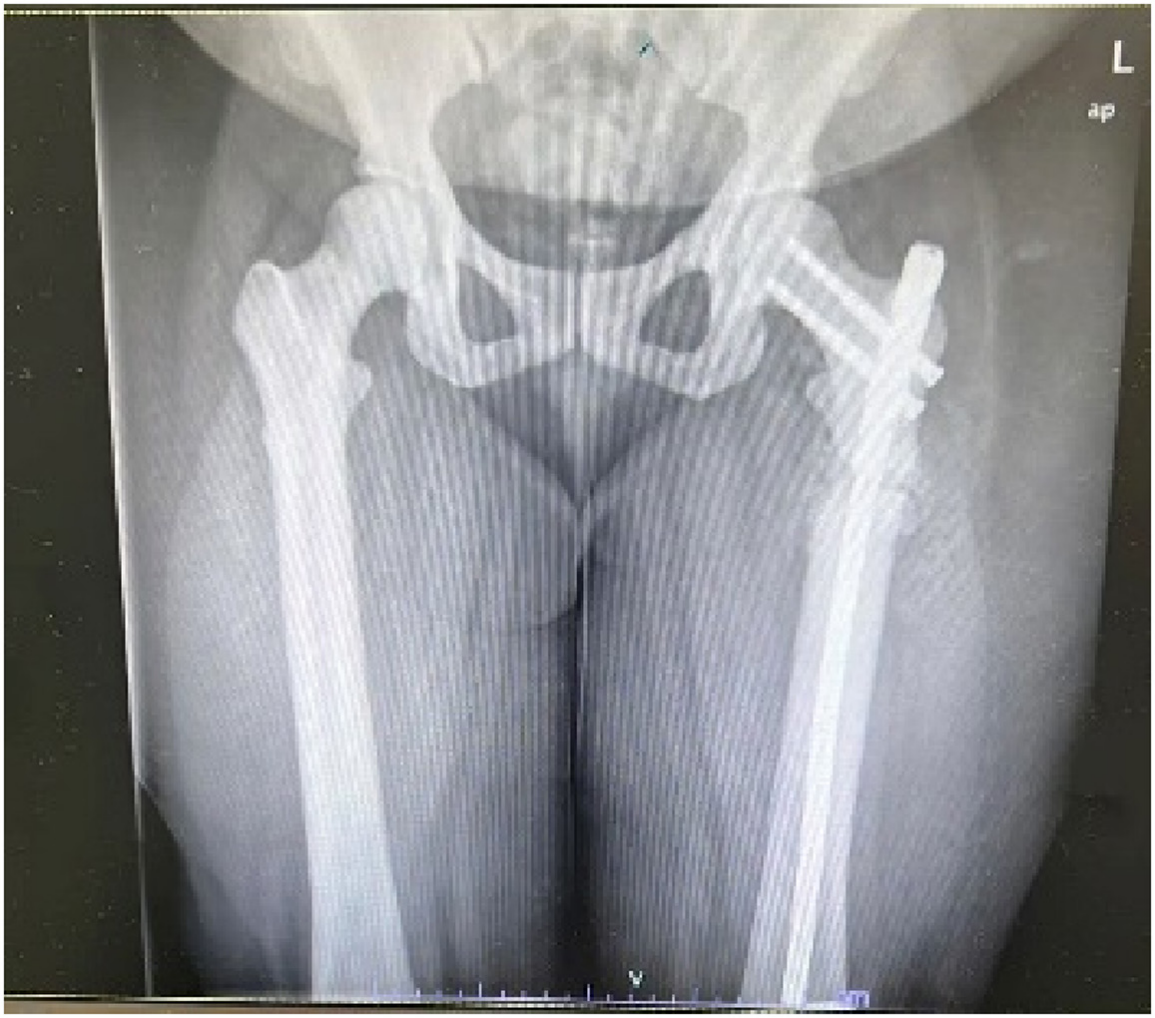
bilateral femur X‐Ray showing bilateral femur pathological fractures—closed reduction of the left femur

She had a history of the bilateral femur pathological fractures (see Figure 2) and underwent closed reduction for the intramedullary left femur early in 2020, under spinal anesthesia.

In addition, the patient had a history of severe obstructive sleep apnea (Apnea Hypopnea Index 1) since 2014 and mild asthma since childhood where she was advised to be on a continuous positive airway pressure (CPAP), but she refused. Furthermore, she had a family history of the same disease in maternal and paternal cousins (familial), dwarfism, her sister, and her maternal grandmother.

Spine x‐ray revealed a multilevel disc dehydration; multiple disc bulges were identified at multiple levels starting in the cervical and lumbar spine. The most marked changes were seen at the C5‐C6 level, where there were left posterior disc bulges causing indentation over the thecal sac with mild narrowing of the central canal.

The patient was referred to a consultant anesthesiologist; a spinal anesthesia was indicated. She delivered a baby boy (weight: 1920 gm, APGAR: 8/10, cord pH 7.30) by breech extraction uterus has a septum inside like 2 cm long and more to the anterior wall, estimated blood loss (EBL) 500 ml.

After operation, patient disconnected from Non‐invasive ventilation (NIV) and continuous positive airway pressure(CPAP), and put on 5 liters O2 via simple facemask and to be transferred to high dependent unit, prior to transfer, ABG done and with normal result (PH 7.39, PCO2 46.2, PO2 114, HCO3 26.9, SO2 99.5). Fifteen minutes after transfer the patient, patient reconnected back to NIV with the same mode of CPAP +8 cm H2O and Fio2 reduced to 30% from 35%, plan for overnight NIV as the patient is known case of OSA, during daytime NIV as indicated especially when patient is sleeping.

**FIGURE 3:**
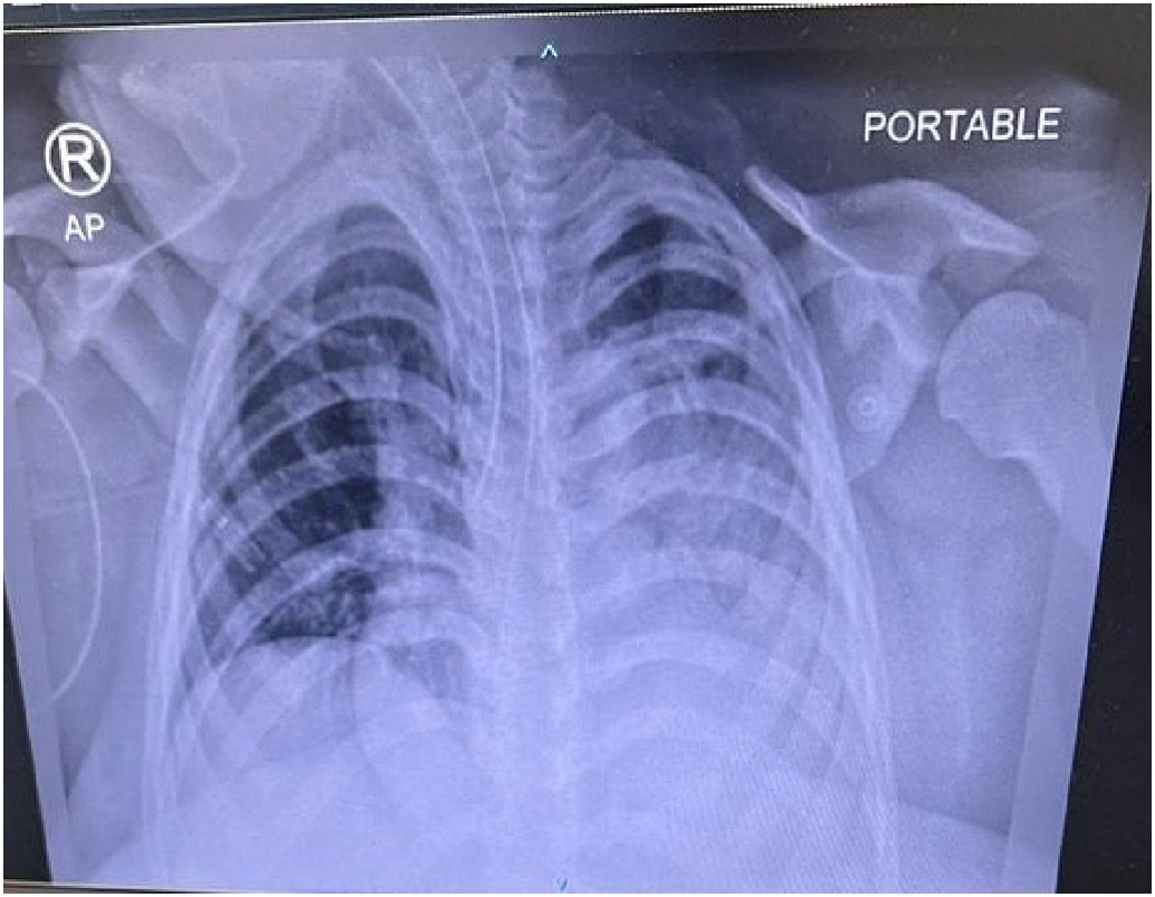
Chest X‐Ray shows endotracheal tube, right fracture at midshaft.

On the day following the lower segment cesarean section (LSCS), she developed cough, palpitation, and tachycardia; the patient desaturated and the oxygen demand gradually increased from 3 L to 8 L/min, then she had a sustained cardiac arrest, cardiopulmonary resuscitation (CPR) was performed for 2 minutes, with 1 dose of adrenaline was given. Later, she developed multiple comorbid cardiac arrests and intubated for 4 days (see Figure 3), then extubated and stepped down to the inpatient ward.

During her ICU stay, she developed multimyoclonus jerking, both upper arm spasms with upward rolling of eyes due to paroxysmal autonomic instability with dystonia (PAID) syndrome. ECG showed sinus tachycardia, T‐wave inversion in leads V1, V2, V3, V4, previous ECG dated back to 2022 showed T‐wave inversions in leads V1 and V2. At the return of spontaneous circulation (ROSC), an arterial blood gas shows PCO2:70, after 10 min of intubation. Repeated ABG showed lactate: 2.4, PCO2: 45, PO2:65 ON 50% FIO2. EEG shows no intracranial mass effect, hemorrhage, or gross ischemic infarction area.

She was seen by a neurologist where EEG was performed and revealed a paroxysmal nonepileptic event and mild–moderate diffuse encephalopathy secondary to sedatives. Few lower extremity jerks were induced only with stimulation recorded with no EEG correlates. A whole spine MRI revealed multi‐disc disease with no other significant findings. Computed tomography arterial portography (CTAP) reported bilateral lower lobes consolidation, more on the left side and bilateral lung changes, which could be related to atelectasis.

The patient showed an improvement regarding her myoclonic movements, affecting her lower limb mainly occur whenever she takes her clonazepam and worsens during evening time (in between the doses). She was unable to sit without support, unable to mobilize, and difficult to hold her legs in flexed posture or against gravity due to myoclonus. The patient still has involuntary movement of the lower part of her body. On the other hand, she is alert, oriented, and speech clear and appropriate. The discharge plan includes providing a wheelchair with a Zimmer frame, home CPAP machine, and home exercises' instructions.

## 3 DISCUSSION

Pycnodysostosis is a rare genetic disorder associated with short limbs, an obtuse mandibular angle, and delayed closure of the cranial sutures. Some individuals also have dental and nail anomalies. Intelligence is usually normal, with only minor psychomotor impairments. These characteristics may make anesthesia management more difficult.^5^ Both sexes are equally affected by the disorder. It is caused by mutations in the gene cathepsin K located on chromosome 1, with an estimated prevalence of 1–3 per 1,000,000.^4^

Pycnodysostosis is caused by a gene that was not discovered until 1995 when relevance analysis studies found on chromosome 1q21, which causes a mutation in cathepsin K, a lysosomal cysteine protease that is highly expressed in osteoclasts, leading to osteosclerosis and impaired bone resorption.^6^

Scoliosis, kyphosis, and lumbar hyperlordosis are spine features of pycnodysostosis.^7^ Our patient had spine revealed multilevel disc dehydration; multiple disc bulges are identified at multiple levels starting in the cervical and lumbar spine. The most marked changes are seen at the C5‐C6 level, where there are left posterior disc bulges causing indentation over the thecal sac with mild narrowing of the central canal. Our patient underwent general anesthesia, though many researchers recommended performing the cesarean section for patients with pycnodysostosis under neuraxial anesthesia.^8^

In summary, we present a case of a pycnodysostosis patient having elective cesarean birth under general anesthesia, who had a cardiac arrest after the operation, resuscitated for 4 days then extubated and shifted to the intensive care unit; later on, she developed Myoclonus jerking impression of the EEG indicative of paroxysmal nonepileptic events and mild–moderate diffuse encephalopathy secondary to sedative medication. Few lower extremity jerks were induced only with stimulation recorded with no EEG correlates. Currently, the patient discharged with CPAP is recommended to follow up with the physiotherapist and psychiatrist.

Several articles supporting that lumbar neuraxial technique is the anesthesia of choice, because of low risk of airway manipulation, hypotension, and uteroplacental hypo perfusion.^1^, ^4^

## AUTHOR CONTRIBUTIONS

AA was involved in the conception and design, the drafting of the paper. AJN & HAB were involved in the data collection, revising it critically for intellectual content; and the final approval of the version to be published. All authors agree to be accountable for all aspects of the work.

## FUNDING INFORMATION

This study was not funded.

## CONFLICT OF INTEREST

The authors declare that they have no competing interests.

## ETHICS APPROVAL

The article describes a case report. Therefore, no additional permission from our Ethics Committee was required.

## CONSENT

Written informed consent was obtained from the patient to publish this report in accordance with the journal's patient consent policy.

## Data Availability

All data generated or analyzed during this study are included in this published article.
